# How do discrepancies between self‐rated and physical health predict self‐reported overnight hospitalizations in later life?

**DOI:** 10.1111/bjhp.70102

**Published:** 2026-08-17

**Authors:** Bill Calvey, James Laurence

**Affiliations:** ^1^ Social Research Institute, University College London London UK

**Keywords:** health and retirement study, health asymmetry, health congruence, overnight hospitalizations, physical health, self‐rated health

## Abstract

**Objectives:**

Discrepancies between self‐rated health (SH) and physical health (PH) are linked to physical and psychological health outcomes in older adults. We investigate whether such discrepancies are also associated with self‐reported overnight hospitalizations.

**Design:**

A prospective, observational cohort design.

**Methods:**

We followed 4373 older adults aged ≥60 years over a 6‐year period in the Health and Retirement Study (2016–2022). Health asymmetry scores were computed by regressing baseline SH onto PH and extracting the residuals, reflecting the degree to which SH deviates from PH. Zero‐inflated negative binomial mixed models, including a random intercept term, tested whether health asymmetry scores predicted (1) any overnight hospitalization and (2) the cumulative number of nights spent in hospital, adjusting for SH, PH and covariates.

**Results:**

Over a 6‐year follow‐up, 55.34% of participants self‐reported at least one overnight hospital stay. Higher health asymmetry scores (i.e., greater health optimism, where SH exceeded PH) were significantly associated with a lower expected number of nights spent in hospital (incidence ratio rate: 0.80; 95% confidence interval: 0.72, 0.89; *p* < .001), independent of SH and PH. However, health asymmetry was not significantly associated with the probability of belonging to the ‘never hospitalized’ group, in the zero‐inflation component.

**Conclusions:**

Discrepancies between SH and PH predict the intensity of self‐reported hospital use above and beyond SH and PH alone. Although health asymmetry did not distinguish those, who were never hospitalized, older adults' interpretations of their health still carry prognostic value and may represent an important psychosocial determinant of health care utilization.

**Statement of Contribution**

**What Is Already Known on this Subject?**

- Health asymmetry captures discrepancies between subjective and objective health in older adults.
- Such discrepancies are associated with physical, functional and psychological health outcomes.
- However, it remains unclear how health asymmetry relates to health care utilization in later life.

**What Does this Study Add?**

- Health asymmetry predicts self‐reported number of hospital nights over a 6‐year period.
- Health optimism is linked with fewer cumulative hospital nights.
- Health asymmetry adds prognostic value beyond what self‐rated and physical health explain.

## INTRODUCTION

Subjective or self‐rated health (SH) is typically measured with a single‐item question, asking respondents to evaluate their overall health (for example, on a scale from ‘poor’ to ‘excellent’). SH is routinely included in major health and ageing studies, due to its robust prognostic utility and brevity. SH is an independent predictor of mortality and morbidity (DeSalvo et al., 2006; Hetlevik et al., 2020) and is also associated with health care utilization, psychological well‐being and functional health in later life (Banerjee et al., 2010; French et al., 2012; Hirosaki et al., 2017; Leng et al., 2025). Importantly, SH is not only a proxy for clinically determined, objective health (OH) status to some degree, but it also captures subjective interpretations of somatic symptoms, fatigue, affect, health beliefs and social comparisons (Bailis et al., 2003). However, to understand why SH is prognostically meaningful, it is necessary to consider how respondents provide these subjective evaluations.

Jylhä's (2009) unified conceptual model provides a theoretical account of this process. In this framework, SH arises from an active cognitive appraisal process in which respondents integrate multiple sources of health‐relevant information (e.g., bodily symptoms, functional limitations, mental state, prior health experiences) and interpret these within their personal and social context. Importantly, the model posits that individuals have access to internal bodily signals, such as subtle symptoms or physiological dysregulations that may not be captured by OH measures. These inputs are synthesized through biopsychosocial pathways into a single self‐evaluation of health. As a result, SH seems to reflect both an individual's interpretation of their underlying biological state and their lived subjective health experience, helping to explain its strong association with later health outcomes, even after accounting for OH.

Despite SH's predictive utility, there has traditionally been a hegemony of OH measures (i.e., diagnostic tests, clinical observations, physical performance tests) over SH assessments in clinical practice (Bourne, 2009). This biomedical emphasis often comes at the expense of understanding a patient's lived experience of health, including self‐reported symptoms, feelings and personal evaluations of well‐being (Bridges et al., 2011; Slevin et al., 1988). However, evidence suggests that, in some cases, SH can reflect a greater sensitivity than OH status (Bailis et al., 2003). For example, SH has been shown to predict mortality in the short‐term to a similar, or sometimes even greater, extent than OH (Idler & Kasl, 1991). In a Finnish study, poor SH was associated with nearly an eightfold risk of mortality over a 5‐year period, compared to a fourfold risk for poor OH (Wuorela et al., 2020). In other cases, SH has also been shown to be a stronger predictor of hospitalization and institutionalization than OH status (Tavenier et al., 2022; Thygesen et al., 2009; Viljanen et al., 2021).

Furthermore, a growing body of research indicates that psychosocial factors underlying SH strongly influence a range of health outcomes, including patterns of health care utilization. For example, patients with higher levels of health confidence spend, on average, 1.5 days less in hospital than those with low confidence (Brown et al., 2024). Similarly, a recent systematic review found that higher in hospital stress significantly worsened patient health and was linked to a prolonged length of stay (Ford et al., 2023). Conversely, older adults who hold positive self‐perceptions of ageing tend to experience fewer and shorter hospitalizations without higher readmission rates (Nakamura et al., 2022; Sun et al., 2017), independent of OH, which suggests genuine improvements in recovery rather than premature discharge. Meanwhile, those with greater dispositional optimism generally recover faster and experience fewer post‐surgical complications (Ai et al., 2024; Alzahrani, 2021).

If such psychosocial processes influence health care use, then discrepancies between an individual's SH and OH may also represent a key determinant of later life service use. While SH and measures of OH are moderately correlated in older adults (Pinquart, 2001), early gerontologists noted some inconsistencies between these indicators (Maddox & Douglass, 1973; Mossey & Shapiro, 1982), with some individuals rating their health in ways that paradoxically diverge from clinical assessments. These discrepancies tend to widen with age, as SH and OH follow distinct trajectories across the life course. To conceptualize these differences, Chipperfield (1993) proposed the *health congruence* framework, which originally compared SH scores (an estimation of self‐rated health) to objectively measured physical health (PH) scores (originally based on a simple count of diseases and chronic illnesses) in older adults. Based on the level of agreement between these two scores, four groups are usually identified: ‘health optimists’ (those who provide positive SH estimations despite poor PH), ‘health pessimists’ (those who rate their SH as poor despite good PH), ‘good health realists’ (those whose good SH aligns with their good PH score) and ‘poor health realists’ (those whose poor SH aligns with their poor PH score).

Findings from this line of work suggest that health optimism confers benefits for longevity, psychological health and functional health (Chipperfield, 1993; Hong et al., 2004). Conversely, health pessimism is associated with declines in physical and psychological well‐being (Ruthig et al., 2011; Ruthig & Chipperfield, 2007). More recently, this concept has evolved into the *health asymmetry* framework, which quantifies the magnitude and direction of the discrepancy between self‐rated and objective measures of health using continuous discrepancy scores (Calvey et al., 2022). Health asymmetry derives a discrepancy score between self‐rated and objectively measured health, which allows for more flexibility and sensitivity in analyses, as opposed to 2 × 2 indicator variables. Health asymmetry has been shown to longitudinally predict future cases of mortality, depressive symptoms and injurious falls in older adults (Calvey et al., 2023; Calvey, Power, Maguire, de Andrade Moral, & de de Andrade Moral, 2024; Calvey, Power, Maguire, Welmer, & Calderón‐Larrañaga, 2024).

However, the extent to which discrepancies between SH and PH predict overnight hospitalizations remains limited and inconsistent. Ruthig and Chipperfield (2007) reported that health pessimists experienced both more frequent and longer hospital admissions than health realists, while health optimists did not significantly differ from realists. However, this study was limited by a small sample size and analytical approaches that did not directly account for the zero‐inflated distribution of hospital admissions. Viljanen et al. (2021) also found that those with both poor SH and PH (i.e., poor health realists) had a significantly higher risk of being institutionalized after a 10‐ and 18‐year follow‐up period, compared to good health realists. Both studies however, measured PH based solely on disease count, which ignores the multidimensionality of health in later life (Cacioppo & Berntson, 2011; Leonardi, 2018; Shaw & Mackinnon, 2004).

Given the rapidly ageing population and increasing demands on health care systems, identifying diverse predictors of hospitalization risk beyond traditional clinical indicators is essential (Hansén et al., 2025). Understanding whether and how health asymmetry influences hospitalization outcomes could provide clinicians with valuable insights into which older adults are at greatest risk of recurrent hospital stays. Therefore, the present study investigates whether discrepancies between SH and objectively measured PH are associated with the self‐reported cumulative count of overnight hospitalizations, among older adults living in the United States. Based on extant literature, we hypothesize that positive discrepancies between SH and PH (i.e., health optimism) will be longitudinally associated with fewer overnight hospitalizations above and beyond SH and PH alone, whereas negative discrepancies (i.e., health pessimism) will be associated with a higher count of self‐reported overnight hospitalizations. Given that discrepancies between SH and PH vary by both age and sex (Calvey, Power, Maguire, de Andrade Moral, & de de Andrade Moral, 2024), we also test whether age and sex significantly moderate the relationship between health asymmetry and self‐reported overnight hospitalizations.

## MATERIALS AND METHODS

### Study population

The Health and Retirement Study (HRS) is an ongoing, nationally representative, population‐based panel study of community‐dwelling American adults aged 50 years and older and their spouses (Juster & Suzman, 1995). HRS collects longitudinal data biennially on the economic and health consequences of transitioning from work to retirement. The initial cohort was interviewed in face‐to‐face surveys in 1992, with follow‐up interviews occurring every 2 years thereafter via telephone or mail. Beginning in 2006, the HRS randomly selected half of the sample to complete enhanced face‐to‐face interviews incorporating physical performance tests, anthropometric measurements and self‐administered psychosocial questionnaires. The remaining half received this enhanced protocol in 2008 on a rotating basis thereafter. All participants (or approved proxy respondents) provided informed consent prior to participation and HRS makes de‐identified data publicly available for secondary analysis.

**FIGURE 1:**
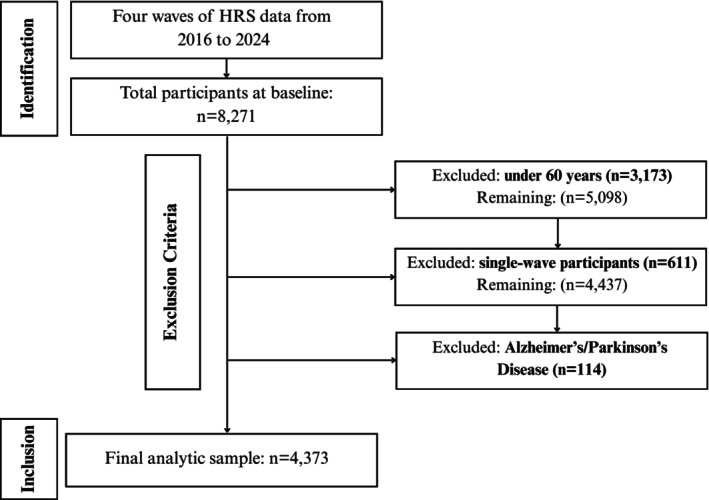
Flow diagram detailing the exclusion criteria that led to the final analytic sample of HRS participants in our study (*n* = 4373).

We analysed 4 consecutive waves of HRS data spanning from 2016 to 2022. From the initial 8271 participants who completed enhanced face‐to‐face interviews in 2016, we removed those from analyses who were under 60 years old (*n* = 3173), to maintain consistency with recent health asymmetry and health congruence research (e.g., Abma et al., 2021; Calvey, Power, Maguire, Welmer, & Calderón‐Larrañaga, 2024). We also excluded those who only participated in one of the 4 HRS waves of interest (*n* = 611), because we could not analyse their outcomes longitudinally. Furthermore, we excluded participants who presented with a formal diagnosis of Alzheimer's Disease or any form of Dementia at baseline (*n* = 114), as the cognitive impairment associated with these conditions may compromise the reliability of SH appraisals and the self‐reporting of overnight hospitalizations. Ultimately, this yielded a final analytic sample of 4373 individuals (see Figure 1). The analytic sample size was determined by the availability of eligible participants within HRS who completed the relevant enhanced face‐to‐face interview waves and met inclusion criteria. Although no a priori power calculation was conducted due to the secondary nature of the data, the large sample size and longitudinal structure provide adequate statistical precision for our analytic approach (Zhang & Yi, 2020), described below.

Participants excluded from the analysis (*n* = 725; aged ≥ 60 years) reported worse SH (*p* < .001; Cohen's d = .48), worse PH (*p* < .001; Cohen's d = .46), were older (*p* < .001; Cohen's d = .46), had fewer years of education (*p* < .001; Cohen's d = .22), reported higher levels of depressive symptoms (*p* < .001; Cohen's d = .31), were more likely to be male (*p* < .001; Cramer's V = .05) and more likely to be a widow (*p* < .001; Cramer's V = .08), compared with those who were included in our study (*n* = 4373). Excluded participants did not differ from included participants in terms of the overall count of overnight hospitalizations or health asymmetry scores. Participant retention across waves was as follows: *n* = 4373 in 2016 (baseline), *n* = 4176 in 2018, *n* = 3630 in 2020 and *n* = 3057 in 2022. We ensured that HRS refreshment cohorts were not included in our analyses. Attrition analyses indicated that longitudinal dropout was associated with several baseline characteristics, including older age, fewer years in education, higher depressive symptoms, poorer SH and more frequent hospital use. However, dropout was not associated with health asymmetry, suggesting that selective attrition is unlikely to bias associations involving the primary exposure (see Data S1).

### Measures

#### Outcome: All‐cause overnight hospitalizations

The primary outcome of interest was the self‐reported number of nights spent in hospital for any cause, over each 2‐year interval. Participants were asked: ‘How many different times were you a patient in a hospital overnight since the last HRS interview date or within the last two years?’ This measure reflects the cumulative number of nights spent in hospital during each period between HRS waves, summing the number of stays across all hospital admissions. Responses were provided biannually at each HRS wave, producing up to four repeated count observations per participant and were modelled as a zero‐inflated count outcome.

#### Exposure: Self‐rated health

At baseline, a single‐item measure captured participants' holistic appraisal of their overall health status. In HRS, participants responded to ‘Would you say your health is excellent, very good, good, fair, or poor?’ on a 5‐point Likert scale. Scores were reverse‐coded so that higher values (e.g., excellent = 5) indicated better SH compared to lower values (e.g., poor =1). SH demonstrates moderate to good test–retest reliability in previous studies (Zajacova & Dowd, 2011), though evidence regarding its agreement with objective health measures is mixed (Araújo et al., 2018; Pinquart, 2001; Wu et al., 2013).

#### Exposure: Physical health

A latent PH score was derived from four clinical indicators measured at baseline: peak expiratory flow, grip strength, walking speed and a count of chronic conditions. Peak expiratory flow was measured three times using a standard range peak flow meter with a disposable mouthpiece, with the averaged score across all three attempts used as the final score. Grip strength was assessed with a Smedley's® spring‐type hand dynamometer, with two measurements taken on the dominant hand; the mean score (kg) was retained. Walking speed was measured in participants' homes over a 98.5‐inch course, during which they were instructed to walk at their usual pace (Maharani et al., 2020), with two‐timed trials averaged to obtain speed in metres per second. Lastly, participants self‐reported whether a doctor had ever told them they had acute or chronic conditions, such as stroke, high blood pressure, diabetes, lung disease, heart problems, cancer and/or arthritis. A simple count of the number of acute or chronic conditions was summed up per participant.

Since peak expiratory flow, grip strength and walking speed vary systematically by sex and height, these three objectively measured PH scores were regressed separately onto sex and height, and the resulting sex‐ and height‐adjusted residuals were extracted. These adjusted residuals, along with a count of chronic conditions, served as indicators in an exploratory factor analysis. One latent PH factor was extracted (SS loading: 0.95; proportion of variance explained = 0.24). Individual standardized loadings on the PH factor were 0.56 (peak expiratory flow), 0.64 (grip strength), 0.28 (walking speed) and 0.33 (chronic conditions). Model fit was acceptable (RMSR = 0.02; RMSEA = 0.036, Tucker–Lewis Index = 0.972). Factor scores were scaled to range from 1 (poor physical health) to 10 (excellent physical health). This latent variable approach has been employed in previous investigations (Calvey, Power, Maguire, de Andrade Moral, & de de Andrade Moral, 2024).

#### Exposure: Health asymmetry

Health asymmetry quantifies discrepancies between SH and objectively measured health in older adults. We used a continuous, zero‐centred discrepancy measure derived from regressing SH onto PH and extracting the residuals, which produced their baseline health asymmetry score. This approach produces residual health asymmetry scores, where positive residuals indicate that an individual's SH exceeds what would be predicted by PH (i.e., health optimistic), negative residuals indicate that respondent's SH was worse than predicted by PH (i.e., health pessimistic), and values near zero indicate alignment between SH and PH assessments (i.e., health realistic). This residual‐based method accounts for the natural correlation between SH and PH while providing a continuous measure of health asymmetry that is analogous to approaches used in health economics literature (Chaparro et al., 2019; Gorman & Sivaganesan, 2007; Johnston et al., 2009). This approach improves on previous studies, which derive a 2 × 2 ordinal categorical variable, which is known to compromise statistical power and flexibility (Calvey et al., 2022; McHugh et al., 2017).

### Statistical analysis

All analyses were conducted in R Studio. There were some missing data points across the entire sample (4.74%). Little's Missing Completely at Random test indicated that the missing data were unlikely to be missing completely at random (*p* < .001). Patterns of missingness were explored using the *naniar* R package (Tierney et al., 2019), which indicated that missing data were most prevalent in the walking speed test. Such missingness was explained by our other covariates and missing variables (i.e., age, sex and weight), consistent with the ‘missing at random’ assumption. Therefore, we imputed this missingness using the R package *Multiple Imputation by Chained Equations* (Van Buuren & Groothuis‐Oudshoorn, 2011). Continuous data were imputed using predictive mean matching while categorical data were imputed via polytomous or logistic regression, where relevant. Following current methodological recommendations to use a sufficient number of imputations (i.e., a number greater than the overall % of missingness) to reduce Monte Carlo error and improve efficiency (e.g., Blomberg & Todorov, 2025; Von Hippel, 2020), 20 imputed datasets were generated with 20 iterations of the imputation algorithm, with results combined into one final imputed dataset using Rubin's rules.

Baseline characteristics were derived for the sample, before and after multiple imputation. Then, to determine whether health asymmetry was associated with self‐reported overnight hospitalizations over a 6‐year follow‐up, a set of Zero‐inflated negative binomial mixed‐effects regression models (ZINBMMs) was conducted. Count data from health care settings frequently exhibit two key challenges: zero‐inflation and overdispersion (Fernandez & Vatcheva, 2022). Zero‐inflation occurs when the observed data contain substantially more zero observations than expected under a standard Poisson distribution, which assumes the mean and variance are approximately equal. This excess of zeros artificially deflates the mean while inflating the variance, potentially biasing parameter estimates and standard errors if left unaddressed (Moghimbeigi et al., 2008). Additionally, overdispersion occurs when the observed variance exceeds that expected by a Poisson model, reflecting additional heterogeneity in the data beyond what the mean alone captures. In our case, the distribution of overnight hospitalization counts across the 6‐year follow‐up exhibited both zero‐inflation and overdispersion, necessitating a more flexible approach than traditional regression models (Yau et al., 2003).

ZINBMMs were therefore selected to examine the association between health asymmetry and self‐reported overnight hospitalizations. These models account for the zero‐inflated and skewed distribution of the overnight hospital count data, which allow for the modelling of random effects in a multi‐level modelling framework. This model estimates a two‐part process simultaneously: a count component models the expected rate of hospitalizations among the ‘at‐risk’ group, and a zero‐response component models the odds of belonging to the excess zero (i.e., ‘never hospitalized’) group. All models included a random intercept per participant, to account for within‐participant clustering across the repeated measurements, allowing each participant to have a distinct baseline level of hospitalization risk, while sharing fixed effect coefficients across the sample. ZINBMMs also produce a dispersion parameter, which indicates the extent of overdispersion within the sample. A score of > 1 indicates the appropriateness of the negative binomial specification over a simple Poisson model. Continuous covariates were standardized (converted into Z‐scores) to aid model convergence, given that large differences in scales can impair numerical stability in ZINBMMs.

A series of sequential models were fitted to isolate the association between health asymmetry and overnight hospitalization counts, while adjusting for age, sex, marital status, education, depressive symptoms (as captured by the CESD) and whether participants are covered by Medicare Insurance. Model 1 examined the association between baseline SH appraisals (dichotomized as ‘good’ or ‘poor’) and overnight hospitalizations. Model 2 tested the association between PH and overnight hospitalizations (with PH also dichotomized into ‘good’ and ‘poor’ PH, based on a population median cut‐off). Model 3 tested an interaction term between SH and PH. SH and PH in these models were dichotomized into ‘good’ and ‘poor’ to address violations of the linearity assumption and to achieve stable model convergence.

Model 4 introduced health asymmetry scores as the primary exposure of interest, without adjusting for SH and PH. Finally, Model 5 (our main model) included health asymmetry scores, while simultaneously controlling for both SH and PH categories, allowing us to examine the independent association between health asymmetry and overnight hospitalizations, above and beyond what SH and PH explain alone (Full models in Data S2). We additionally conducted a sensitivity analysis, which excluded any participant who presented with an overnight hospital stay 2 years prior to baseline, as an attempt to mitigate potential reverse causality bias (See Data S3). We also modelled health asymmetry as a quadratic term, given that the relationship between health asymmetry and hospitalizations may be non‐linear (See Data S4). Finally, we included age‐ and sex‐related interaction terms with health asymmetry, to determine whether age and sex modify the relationship between health asymmetry and cumulative nights spent in hospital (See Data S5). We used the R package *GLMMadaptive* (Rizopoulos, 2020) to carry out our analyses and the R script that supports these findings can be found in Data S6.

## RESULTS

In a sample of 4373 older American adults, 2420 individuals (55.34%) self‐reported at least one overnight stay in a hospital over a 6‐year follow‐up. The mean age of the sample at baseline was 71.4 (±8.39), with 59.62% of the sample being female. The mean SH score was 3.15 (±1.00), while the mean PH score was 5.51 (±0.85) (see Table 1 below for full descriptive statistics). There was a significant positive correlation between SH and PH in our sample (*r*(4371) = .36, *p* < .001). We visualized the distribution of SH, PH and health asymmetry scores in Data S7.

**TABLE 1:** Baseline characteristics of our sample of older American adults from the Health and Retirement Study (*n* = 4373), before and after multiple imputation.

	Before imputation		After imputation
	% (*n*)/mean (SD)	Missing (*n*)	% (*n*)/mean (SD)
Age	71.40 (8.39)	0	71.40 (8.39)
Sex		0	
Males	40.38% (*n* = 1766)		40.38% (*n* = 1766)
Females	59.62% (*n* = 2607)		59.62% (*n* = 2607)
Education (in years)	12.92 (3.14)	57	12.92 (3.14)
Marital status		11	
Married	56.62% (*n* = 2476)		56.83% (*n* = 2485)
Single/Divorced	22.02% (*n* = 963)		22.04% (*n* = 964)
Widowed	21.10% (*n* = 923)		21.13% (*n* = 924)
Depressive symptoms	2.92 (1.31)	0	2.92 (1.31)
Medicare Health Insurance		0	
Yes	73.86% (*n* = 3230)		73.86% (*n* = 3230)
No	26.14% (*n* = 1143)		26.14% (*n* = 1143)
Expiratory flow	327.37 (133.32)	47	327.11 (133.28)
Grip strength	25.9 (9.71)	156	26 (9.73)
Walking speed	3.7 (7.79)	1431	3.81 (7.62)
Count of chronic conditions	2.51 (1.56)	0	2.51 (1.56)
Self‐rated health	3.15 (1.01)	5	3.15 (1)
Cumulative nights spent in hospital	0.45 (1.08)	0	0.45 (1.08)

To determine how health asymmetry scores were associated with overnight hospitalizations, we ran a set of ZINBMMs. All models accounted for the significant zero‐inflation (65% across all four waves) and the non‐independence of repeated measures via a participant‐level random intercept. In model 1, we examined the effect of SH on self‐reported hospitalization risk. Older adults reporting a poor SH appraisal had an expected 2.26 times higher count of overnight hospitalizations [Incidence Rate Ratio (IRR): 2.26; 95% Confidence Intervals (CI): 1.98, 2.58; *p* < .001], compared to those with good SH. In the zero‐component, poor SH was not significantly associated with belonging to the ‘never hospitalized’ group, than those with good SH (Odds Ratio (OR): 0.93; 95% CI: 0.59, 1.47; *p* > .05) (See Data S2). Similarly in model 2, those with a poor PH score had an expected 1.93 times higher count of overnight hospitalizations (IRR: 1.93; 95% CI: 1.69, 2.21; *p* < .001), compared to those with good PH. Meanwhile, poor PH was also not significantly associated with belonging to the ‘never hospitalized’ group (See Data S2). Model 3 tested an interaction term between SH and PH scores in the same model; however, no significant interaction between SH and PH was observed in both the count‐response and zero‐response components (see Data S2).

Model 4 tested the association between health asymmetry scores and overnight hospitalizations, without adjusting for individual‐level effects of SH or PH. We found that for every 1‐unit increase in health asymmetry (i.e., becoming increasingly health optimistic), the expected count of overnight hospitalizations decreased by 30% over the study period (IRR: 0.70; 95% CI: 0.65, 0.75; *p* < .001). Concurrently, there was no statistically significant increase in odds of belonging to the ‘never hospitalized’ group as determined by health asymmetry scores (See Data S2).

In our final ZINBMM (see Table 2 below), we simultaneously examined the independent association of health asymmetry scores on the cumulative count of overnight hospitalizations, while controlling for the individual effects of SH and PH. The model successfully accounted for the complex data structure, with a considerable proportion of the observed data points being zeros (65%). A dispersion parameter of 2.28 indicated substantial overdispersion, confirming the choice of a negative binomial rather than a Poisson specification. The estimated variance for the random intercept term was 1.02, indicating there was unexplained individual‐level heterogeneity in the baseline self‐reported hospitalization rates across our sample of older adults. For every 1‐unit increase in health asymmetry (i.e., becoming increasingly health optimistic), a 20% decrease in the cumulative number of nights spent in hospital over the study period was observed (IRR: 0.80; 95% CI: 0.72, 0.89; *p* < .001), after controlling for fixed effects, including SH and PH. Meanwhile, there was no statistically significant association between health asymmetry and belonging to the ‘never hospitalized’ in the zero‐response component.

**TABLE 2:** Zero‐inflated negative binomial mixed‐effects model for overdispersed counts of self‐reported overnight hospitalizations in a sample of older adults in the United States (*n* = 4373).

	Estimate	IRR	95% CI
Count‐response component			
Time	.12	1.12***	(1.07/1.18)
Age	.10	1.10*	(1.02/1.19)
Sex: Females	−.21	0.81***	(0.72/0.92)
Education (in years)	.04	1.05	(0.97/1.13)
Marital Status: Single/Divorced	.05	1.05	(0.90/1.22)
Marital Status: Widowed	.01	1.05	(0.87/1.17)
Depressive Symptoms	.09	1.09***	(1.03/1.16)
Medicare Health Insurance: No	−.24	0.78**	(0.66/0.94)
Health asymmetry	−.22	0.80***	(0.72/0.89)
Self‐rated health: Poor	.34	1.41***	(1.15/1.72)
Objective health: Poor	.58	1.78***	(1.55/2.05)

	Estimate	OR	95% CI
Zero‐response component			
Time	.24	1.27*	(1.02/1.58)
Age	−.56	0.57**	(0.39/0.84)
Sex: Females	−.12	0.89	(0.58/1.34)
Education (in years)	−.41	0.66***	(0.52/0.85)
Marital status: Single/Divorced	−.21	0.80	(0.48/1.37)
Marital status: Widowed	−.29	0.75	(0.40/1.43)
Depressive symptoms	−.23	0.80	(0.63/1.00)
Medicare Health Insurance: No	.19	1.21	(0.72/2.04)
Health asymmetry	.32	1.37	(0.93/2.03)
Self‐rated health: Poor	.36	1.43	(0.69/2.98)
Objective health: Poor	−.03	0.97	(0.57/1.62)
Model fit and random components			
AIC	25113.05		
BIC	25278.86		
Log‐likelihood	−12530.53		
Random intercept variance	1.02		
Dispersion parameter	2.28		
Frequency (Proportion) of zeros	11,290 (65%)		

*Note*: Full models in Supporting Information. Significant at *p < .05, **p < .01, ***p < .001. All continuous variables were *Z*‐standardized prior to modelling.

Abbreviations: AIC, Akaike information criteria; BIC, Bayesian information criteria; IRR, incidence rate ratio; OR, odds ratio.

To mitigate potential bias from reverse causality, where previous hospitalizations might influence baseline health asymmetry scores, we conducted a sensitivity analysis, which excluded older adults who self‐reported any overnight hospital stays within 2 years prior to our baseline assessment in HRS. This sensitivity ZINBMM found similar estimates to our main findings (See Data S3). Specifically, for every 1‐unit increase in health asymmetry, the expected count of overnight hospitalizations resulted in a 19% decrease in the number of nights spent in hospital over the study period (IRR: 0.81; 95% CI: 0.70, 0.92; *p* < .001). Furthermore, we modelled health asymmetry as a quadratic term (see Data S4) to assess potential non‐linear effects. No evidence of a significant non‐linear association with hospitalization was observed and the results were consistent with the linear specification. Finally, we tested whether age and sex moderated the association between health asymmetry and overnight hospitalizations by including interaction terms in the model. No significant interaction was observed between health asymmetry and sex. However, a significant interaction was found between health asymmetry and age in the zero‐response component of the model (OR = 0.65, 95% CI [0.48, 0.87]). This indicates that the association between health asymmetry and the likelihood of remaining hospitalization‐free varied across age (see Data S5), such that higher health optimism was associated with a lower probability of belonging to the zero‐hospitalization group at older ages, whereas this association was weaker at younger ages.

## DISCUSSION

We investigated whether health asymmetry scores were associated with overnight hospitalizations among older adults over a 6‐year period. Using a population‐based cohort of American older adults, we found that greater health asymmetry scores (i.e., in the direction of health optimism), were longitudinally associated with a reduced expected count of self‐reported overnight hospitalizations. However, health asymmetry was not associated with the likelihood of belonging to the zero‐hospitalization group, indicating that it did not differentiate individuals who remained entirely hospitalization‐free. Importantly, this association between health asymmetry and the cumulative count of overnight hospitalizations remained robust even after controlling for both the individual's SH appraisal and PH status, suggesting that the discrepancy itself constitutes an independent psychological resource. These findings provide novel empirical support for the idea that how older adults perceive and interpret their health, and the discrepancy between this SH estimation and their PH status, carries prognostic value beyond their clinical status and influences tangible health outcomes.

Our findings partially align with prior work on health congruence. Ruthig and Chipperfield (2007) observed that health pessimists experienced more frequent hospital admissions and longer hospital stays than health realists, though they found no significant difference between health optimists and health realists. Viljanen et al. (2021) found that only those with poor SH and PH (i.e., poor health realists) had a significantly elevated risk of institutionalization compared to good health realists, but not health optimists or pessimists. Simon et al. (2025) also found that individuals with no limitations in instrumental activities of daily living despite being in poor SH also had increased institutionalization risk. There is substantial heterogeneity in prior findings, and our results differ from some of these studies, likely due to methodological and conceptual differences. Firstly, we obtained a residual score for each participant based on the discrepancy between SH and PH which preserves statistical power (Altman & Royston, 2006; McHugh et al., 2017), as opposed to creating a 2 × 2 ordinal categorical variable. Secondly, we measured PH multidimensionally, incorporating scores from performance tests alongside disease burden, unlike previous studies that relied solely on disease counts (Ruthig & Chipperfield, 2007; Viljanen et al., 2021).

This association between health asymmetry and the cumulative count of overnight hospitalizations may be explained through several psychological and behavioural pathways. Optimism is consistently linked with adaptive coping, proactive health engagement and resilience under stress (Carver et al., 2010; Rasmussen et al., 2009). As a result, older adults who perceive their health more optimistically may engage more in preventive behaviours, adhere better to medical recommendations and remain physically active (Steptoe et al., 2006), an adaptive mechanism which likely reduces their risk of recurrent hospitalizations. Even following a hospitalization, a health optimistic outlook may promote better compliance with rehabilitation and post‐discharge care, thereby reducing the likelihood of accumulating overnight hospital stays.

However, this should not be interpreted to mean that health optimism is universally beneficial. We found that the relationship between health asymmetry and belonging to the hospitalization‐free group was moderated by age, such that older adults with higher health optimism were less likely to remain free of hospitalizations. One plausible explanation is that this pattern reflects differences in health care‐seeking behaviours. Health optimists may underestimate their risk of illness and consequently under‐report symptoms, delay seeking medical attention and avoid hospital visits where possible (Fragkaki et al., 2021; Park et al., 2014; Wendt, 2005). While such tendencies may reduce health care use earlier in older adulthood, they may ultimately result in greater hospital exposure at more advanced ages when underlying PH declines more rapidly. This interpretation is consistent with prior research showing that health optimists had the highest risk of experiencing an injurious fall (Calvey, Power, Maguire, Welmer, & Calderón‐Larrañaga, 2024), and suggests that promoting a healthy dose of realism may be more appropriate when older adults face age‐related declines in health (Chipperfield et al., 2019).

Conversely, health pessimism has been associated with increased distress, maladaptive coping and more frequent use of health services (Hirschmiller et al., 2025; Ruthig et al., 2011; Ruthig & Chipperfield, 2007). Individuals who perceive their health more negatively may be less inclined to engage in preventive health strategies and more likely to interpret benign bodily sensations as signs of illness. Health pessimism may also overlap with health anxiety (Calvey et al., 2022), a construct which has been linked to excessive health service use. For example, Norbye et al. (2022) found that even small increases in health anxiety predicted greater use of primary, somatic specialist and mental health services. Relatedly, health pessimists who believe that they are in poor OH just might end up in poor OH through different psychological and behavioural pathways (Calvey, Power, Maguire, de Andrade Moral, & de de Andrade Moral, 2024; Idler & Kasl, 1991), and as such, may find themselves in a vicious cycle of illness and hospitalization.

Essentially, a modest degree of health optimism may function as a psychological buffer, promoting well‐being and protecting against excessive and prolonged hospital stays, particularly in earlier stages of older adulthood. Health optimism increases with age (Calvey, Power, Maguire, de Andrade Moral, & de de Andrade Moral, 2024), potentially reflecting self‐protective adaptation or response shifts, processes through which individuals recalibrate their internal standards of health in light of age‐related decline (Sprangers & Schwartz, 1999). From this perspective, health optimism may represent a partially adaptive and prophylactic psychological strategy, as being health optimistic is protective against depressive symptoms, functional decline and mortality in older adults (Borawski et al., 1996; Chipperfield, 1993; Patton et al., 2011). Incongruence in the form of health pessimism, however, may erode well‐being and contribute to greater health care use (Ruthig & Chipperfield, 2007). Thus, being health optimistic, despite being in poorer OH, may provide a long‐term advantage, reducing the recurrent overnight hospitalizations in later life.

Our findings emphasize the prognostic value of a health asymmetry metric. Identifying health asymmetry status in older adults, particularly within community and primary care settings could help clinicians detect individuals at‐risk of future or prolonged hospitalizations. This approach may complement traditional risk stratification methods that rely solely on objective indicators of disease. Conversely, this metric may have limited applicability in acute hospital environments, where decisions are necessarily guided by objective measures and rapid clinical judgements. Beyond its immediate clinical use, the health asymmetry framework enables the prognostication of health outcomes by modelling the interactive and synergistic effects of subjective and objective health scores. This approach offers a richer understanding than considering SH or OH in isolation. Discrepancies between SH and OH have clinical pertinence not only for hospitalization risk, but also for survival, depressive symptomatology and injurious fall risk (Calvey et al., 2023; Calvey, Power, Maguire, de Andrade Moral, & de de Andrade Moral, 2024; Calvey, Power, Maguire, Welmer, & Calderón‐Larrañaga, 2024).

A notable strength of this study is the use of a large, nationally representative sample of American older adults, analysed over an extended 6‐year follow‐up. The use of ZINBMMs is a methodological advancement over prior studies that did not directly account for overdispersed or zero‐inflated hospital data (e.g., Ruthig & Chipperfield, 2007). This approach more accurately captures the real‐world distribution of hospitalization counts, where a substantial proportion of older adults experienced no hospital stays across multiple waves.

Nonetheless, several limitations warrant consideration. Firstly, we relied on self‐reported counts of overnight hospitalizations rather than administrative health records. Self‐reports are susceptible to recall bias and may introduce endogeneity with SH measures. Individuals with generally positive affect may overestimate their health while underreporting hospital events, which could artificially strengthen the observed protective effect of health optimism. Bhandari and Wagner (2006) suggested that the accuracy of recalling health care use is influenced by cognitive function and sample characteristics. To mitigate this, we excluded participants with baseline diagnoses of Alzheimer's disease or other dementias, similar to previous studies (Calvey, Power, Maguire, de Andrade Moral, & de de Andrade Moral, 2024; Shulman et al., 2006). Moreover, a sensitivity analysis excluding participants with recent hospital stays in HRS confirmed that the association between health asymmetry and hospitalization persisted, suggesting that our findings may not be driven by prior hospital experience (i.e., reverse causality).

Secondly, participants excluded due to single‐wave attendance (or other exclusion criteria) differed systematically from the analytic sample in being older, in poorer health and more socially vulnerable. While they did not differ in hospitalization counts or health asymmetry scores, this selective attrition likely resulted in a healthier sample, potentially attenuating observed associations and limiting generalizability to more vulnerable older adults. Attrition bias cannot also be ruled out, given that longitudinal dropout was associated with several baseline characteristics, including our outcome of interest. However, dropout was not associated with health asymmetry itself. Thirdly, although our measure of PH incorporated both performance tests (e.g., peak flow, grip strength, walking speed) and self‐reported chronic conditions via a latent factor analysis, it may still omit relevant medical or functional dimensions associated with hospitalization risk. However, this objective PH approach represents an improvement over previous studies that relied solely on physician ratings, frailty indices and comorbidity counts (Calvey et al., 2023; Hong et al., 2004; Rai et al., 2019).

Our measure of hospitalization did not include day procedures or emergency visits without an overnight stay, which underestimates the true rate of hospital use. Additionally, formal and informal care resources, which are known to influence the risk of institutionalization and hospitalization (Gaugler et al., 2007; Salminen et al., 2020), were not controlled for in this study and could be a potential avenue for future research. Finally, we did not explore the psychosocial pathways that mediate the link between health asymmetry and hospitalizations. Variables such as coping style, perceived control or social support may explain why greater health asymmetry in the direction of health optimism appears partially protective, particularly in earlier stages of older adulthood. Future research could address this.

## CONCLUSION

Overall, discrepancies between SH and PH are associated with the intensity of recurrent overnight hospitalizations among American older adults. These findings underscore the importance of integrating psychosocial constructs like health asymmetry into research and clinical practice when examining hospitalization risk among older adults. The discrepancy between SH and PH among this population constitutes an independent psychological resource which may protect against cumulative counts of overnight hospitalizations in the early stages of older adulthood. In essence, how older adults perceive and interpret their health status carries important prognostic value beyond their PH status and can influence tangible health outcomes.

## AUTHOR CONTRIBUTIONS

**Bill Calvey:** Conceptualization; data curation; formal analysis; visualization; writing – original draft; methodology; investigation; writing – review and editing; validation. **James Laurence:** Conceptualization; formal analysis; writing – review and editing; funding acquisition; investigation; methodology.

## Supporting information


**Data S1.** Binomial logistic regression analysis of baseline characteristics predicting longitudinal dropout.
**Data S2**. Full zero‐inflated negative binomial mixed‐effects models for overdispersed counts of self‐reported overnight hospitalizations in a sample of older adults in the United States (*n* = 4373).
**Data S3**. Zero‐inflated Negative Binomial Mixed‐effects regression models for overdispersed counts of self‐reported overnight hospitalizations in older adults, removing those who had self‐reported an overnight hospital stay 2 years prior to baseline (*n* = 898).
**Data S4**. Zero‐inflated Negative Binomial Mixed‐effects regression models for overdispersed counts of self‐reported overnight hospitalizations in older adults, with health asymmetry modelled as non‐linear (*n* = 4373).
**Data S5**. Zero‐inflated Negative Binomial Mixed‐effects regression models for overdispersed counts of self‐reported overnight hospitalizations in older adults, with age and sex interactions with health asymmetry (*n* = 4373).
**Data S6**. R script to replicate physical health scores and Zero‐inflated Negative Binomial Mixed‐effects models.
**Data S7**. Distribution of subjective health, objective health and health asymmetry scores.

## Data Availability

The data which support the findings are available at (http://hrsonline.isr.umich.edu). The RAND HRS Data file is an open access dataset based on the HRS data. It was developed at RAND with funding from the National Institute on Aging and the Social Security Administration. The data products are available without cost to registered users.
