# Supplementary material for: How do discrepancies between self‐rated and physical health predict self‐reported overnight hospitalizations in later life?
Source: Br J Health Psychol. 2026 Aug 17;31(3):e70102. doi: 10.1111/bjhp.70102 (PMC13479328; doi:10.1111/bjhp.70102)
Supplement: Supplementary file 1 [file BJHP-31-0-s001.docx]

| **Supplementary Materials 1**  Binomial logistic regression analysis of baseline characteristics predicting longitudinal dropout. | | |
| --- | --- | --- |
|  | **OR** | **p** |
| Age | 1.05*** | <.001 |
| Sex: Females | 0.78*** | <.001 |
| Education (in years) | 0.96*** | <.001 |
| Marital Status: Single/Divorced | 1.45*** | <.001 |
| Marital Status: Widowed | 1.12 | .21 |
| Depressive Symptoms | 1.09** | .002 |
| Medicare Health Insurance: No | 0.98 | .08 |
| Self-rated Health: Poor | 1.37* | .01 |
| Objective Health: Poor | 0.72*** | <.001 |
| Cumulative nights in hospital | 1.08* | .01 |
| Health Asymmetry | 0.94 | .26 |
| Notes: OR = Odds Ratio; Significant at ∗*P* < .05, ∗∗*P* < .01, ∗∗∗ *P* < .001. | | |

| **Supplementary Materials 2**  Full zero-inflated negative binomial mixed-effects models for overdispersed counts of self-reported overnight hospitalisations in a sample of older adults in the United States (n=4,373). | | | |
| --- | --- | --- | --- |
|  | **Estimate** | **IRR/OR** | **95% CI** |
| **Model 1** |  |  |  |
| *Count-response component* |  |  |  |
| Time | .10 | 1.12*** | (1.04 / 1.17) |
| Age | .17 | 1.19*** | (1.10 / 1.28) |
| Sex: Females | -.23 | 0.80*** | (0.70 / 0.91) |
| Education (in years) | -.01 | 0.99 | (0.92 / 1.08) |
| Marital Status: Single/Divorced | .12 | 1.13 | (0.97 / 1.31) |
| Marital Status: Widowed | .04 | 1.04 | (0.89 / 1.21) |
| Depressive Symptoms | .12 | 1.13*** | (1.06 / 1.20) |
| Medicare Health Insurance: No | -.28 | 0.75** | (0.63 / 0.90) |
| Self-rated Health: Poor | .82 | 2.26*** | (1.98 / 2.58) |
| *Zero-response component* |  |  |  |
| Time | .22 | 1.25* | (1.01 / 1.55) |
| Age | -.38 | 0.68* | (0.49 / 0.94) |
| Sex: Females | -.15 | 0.86 | (0.56 / 1.32) |
| Education (in years) | -.50 | 0.61*** | (0.47 / 0.78) |
| Marital Status: Single/Divorced | -.09 | 0.91 | (0.54 / 1.53) |
| Marital Status: Widowed | -.24 | 0.79 | (0.42 / 1.48) |
| Depressive Symptoms | -.23 | 0.79 | (0.62 / 1.01) |
| Medicare Health Insurance: No | .29 | 1.33 | (0.77 / 2.31) |
| Self-rated Health: Poor | -.08 | 0.93 | (0.59 / 1.47) |
| *Model fit and random components* |  |  |  |
| AIC | 25251.39 |  |  |
| BIC | 25391.69 |  |  |
| Log-likelihood | -12603.70 |  |  |
| Random Intercept Variance | 1.01 |  |  |
| Dispersion Parameter | 2.27 |  |  |
| **Model 2** |  |  |  |
| *Count-response Component* |  |  |  |
| Time | .11 | 1.12*** | (1.06 / 1.18) |
| Age | .05 | 1.05 | (0.97 / 1.14) |
| Sex: Females | -.22 | 0.80*** | (0.71 / 0.91) |
| Education (in years) | -.02 | 0.97 | (0.89 / 1.06) |
| Marital Status: Single/Divorced | .08 | 1.08 | (0.93 / 1.26) |
| Marital Status: Widowed | .02 | 1.01 | (0.87 / 1.19) |
| Depressive Symptoms | .18 | 1.19*** | (1.12 / 1.27) |
| Medicare Health Insurance: No | -.29 | 0.75** | (0.62 / 0.89) |
| Physical Health: Poor | .66 | 1.93*** | (1.69 / 2.21) |
| *Zero-response component* |  |  |  |
| Time | .24 | 1.27* | (1.01 / 1.60) |
| Age | -.44 | 0.65* | (0.45 / 0.93) |
| Sex: Females | -.15 | 0.86 | (0.56 / 1.34) |
| Education (in years) | -.42 | 0.66** | (0.49 / 0.87) |
| Marital Status: Single/Divorced | -.16 | 0.85 | (0.49 / 1.48) |
| Marital Status: Widowed | -.27 | 0.76 | (0.39 / 1.48) |
| Depressive Symptoms | -.27 | 0.76* | (0.59 / 0.99) |
| Medicare Health Insurance | .27 | 1.31 | (0.76 / 2.26) |
| Physical Health: Poor | .13 | 1.14 | (0.69 / 1.87) |
| *Model fit and random components* |  |  |  |
| AIC | 25406.86 |  |  |
| BIC | 25477.05 |  |  |
| Log-likelihood | -12692.43 |  |  |
| Random Intercept Variance | 1.06 |  |  |
| Dispersion Parameter | 2.27 |  |  |
| **Model 3** |  |  |  |
| *Count-response Component* |  |  |  |
| Time | .12 | 0.25*** | (1.08 / 1.19) |
| Age | .08 | 1.09 | (1.01 / 1.15) |
| Sex: Females | -.22 | 0.81*** | (0.71 / 0.91) |
| Education (in years) | .04 | 1.03 | (0.96 / 1.12) |
| Marital Status: Single/Divorced | .06 | 1.06 | (0.91 / 1.23) |
| Marital Status: Widowed | .04 | 1.01 | (0.90 / 1.21) |
| Depressive Symptoms | .10 | 1.10** | (1.04 / 1.17) |
| Medicare Health Insurance | -.25 | 0.78** | (0.65 / 0.93) |
| Self-rated Health: Poor | .71 | 2.03*** | (1.61 / 2.56) |
| Physical Health: Poor | .55 | 1.69*** | (1.48 / 2.01) |
| Self-rated*Physical Health | -.01 | .99 | (0.76 / 1.30) |
| *Zero-response component* |  |  |  |
| Time | .28 | 1.32* | (1.06 / 1.64) |
| Age | -.46 | 0.63** | (0.46 / 0.87) |
| Sex: Females | -.23 | 0.80 | (0.50 /1.25) |
| Education (in years) | -.43 | 0.65*** | (0.50 / 0.85) |
| Marital Status: Single/Divorced | -.18 | 0.84 | (0.50 / 1.41) |
| Marital Status: Widowed | -.21 | 0.81 | (0.44 / 1.50) |
| Depressive Symptoms | -.22 | 0.80 | (0.63 / 1.01) |
| Medicare Health Insurance | .28 | 1.32 | (0.77 / 2.24) |
| Self-rated Health: Poor | -.34 | 0.71 | (0.30 / 1.68) |
| Physical Health: Poor | .03 | 1.03 | (0.56 / 1.92) |
| Self-rated*Physical Health | .40 | 1.50 | (0.55 / 4.09) |
| *Model fit and random components* |  |  |  |
| AIC | 25144.25 |  |  |
| BIC | 25310.30 |  |  |
| Log-likelihood | -12546.13 |  |  |
| Random Intercept Variance | 0.97 |  |  |
| Dispersion Parameter | 2.24 |  |  |
| **Model 4** |  |  |  |
| *Count-response Component* |  |  |  |
| Time | .11 | 1.11*** | (1.06 / 1.17) |
| Age | .21 | 1.23*** | (1.14 / 1.33) |
| Sex: Females | -.23 | 0.79*** | (0.70 / 0.90) |
| Education (in years) | -.02 | 0.98 | (0.90 / 1.06) |
| Marital Status: Single/Divorced | .15 | 1.16 | (0.99 / 1.35) |
| Marital Status: Widowed | .04 | 1.04 | (0.89 / 1.21) |
| Depressive Symptoms | .15 | 1.16*** | (1.08 / 1.23) |
| Medicare Health Insurance: No | -.30 | 0.74** | (0.61 / 0.89) |
| Health Asymmetry | -.36 | 0.70*** | (0.65 / 0.75) |
| *Zero-response component* |  |  |  |
| Time | .22 | 1.25* | (1.01 / 1.55) |
| Age | -.45 | 0.64* | (0.44 / 0.92) |
| Sex: Females | .11 | 0.90 | (0.59 / 1.37) |
| Education (in years) | -.47 | 0.63*** | (0.49 / 0.80) |
| Marital Status: Single/Divorced | -.11 | 0.89 | (0.53 / 1.49) |
| Marital Status: Widowed | -.27 | 0.76 | (0.39 / 1.47) |
| Depressive Symptoms | -.22 | 0.80 | (0.64 / 1.02) |
| Medicare Health Insurance: No | .23 | 1.25 | (0.73 / 2.17) |
| Health Asymmetry | .11 | 1.12 | (0.87 / 1.44) |
| *Model fit and random components* |  |  |  |
| AIC | 25273.77 |  |  |
| BIC | 25414.06 |  |  |
| Log-likelihood | -12614.89 |  |  |
| Random Intercept Variance | 1.01 |  |  |
| Dispersion Parameter | 2.29 |  |  |
| **Model 5** |  |  |  |
| *Count-response Component* |  |  |  |
| Time | .12 | 1.12*** | (1.07 / 1.18) |
| Age | .10 | 1.10* | (1.02 / 1.19) |
| Sex: Females | -.21 | 0.81*** | (0.72 / 0.92) |
| Education (in years) | .04 | 1.05 | (0.97 / 1.13) |
| Marital Status: Single/Divorced | .05 | 1.05 | (0.90 / 1.22) |
| Marital Status: Widowed | .01 | 1.05 | (0.87 / 1.17) |
| Depressive Symptoms | .09 | 1.09*** | (1.03 / 1.16) |
| Medicare Health Insurance: No | -.24 | 0.78** | (0.66 / 0.94) |
| Health Asymmetry | -.22 | 0.80*** | (0.72 / 0.89) |
| Self-rated Health: Poor | .34 | 1.41*** | (1.15 / 1.72) |
| Objective Health: Poor | .58 | 1.78*** | (1.55 / 2.05) |
| *Zero-response component* |  |  |  |
| Time | .24 | 1.27* | (1.02 / 1.58) |
| Age | -.56 | 0.57** | (0.39 / 0.84) |
| Sex: Females | -.12 | 0.89 | (0.58 / 1.34) |
| Education (in years) | -.41 | 0.66*** | (0.52 / 0.85) |
| Marital Status: Single/Divorced | -.21 | 0.80 | (0.48 / 1.37) |
| Marital Status: Widowed | -.29 | 0.75 | (0.40 / 1.43) |
| Depressive Symptoms | -.23 | 0.80 | (0.63 / 1.00) |
| Medicare Health Insurance: No | .19 | 1.21 | (0.72 / 2.04) |
| Health Asymmetry | .32 | 1.37 | (0.93 / 2.03) |
| Self-rated Health: Poor | .36 | 1.43 | (0.69 / 2.98) |
| Objective Health: Poor | -.03 | 0.97 | (0.57 / 1.62) |
| *Model fit and random components* |  |  |  |
| AIC | 25113.05 |  |  |
| BIC | 25278.86 |  |  |
| Log-likelihood | -12530.53 |  |  |
| Random Intercept Variance | 1.02 |  |  |
| Dispersion Parameter | 2.28 |  |  |
| Frequency (Proportion) of Zeros | 11,290 (65%) |  |  |
| Notes: IRR: Incidence Rate Ratio; OR: Odds Ratio; AIC: Akaike Information Criteria; BIC: Bayesian Information Criteria; Significant at ∗*P* < .05, ∗∗*P* < .01, ∗∗∗ *P* < .001; All continuous variables were Z-standardised prior to modelling. | | | |

| **Supplementary Materials 3**  Zero-inflated Negative Binomial Mixed-effects regression models for overdispersed counts of self-reported overnight hospitalisations in older adults, removing those who had self-reported an **overnight hospital stay 2 years prior to baseline** (n=898). | | | |
| --- | --- | --- | --- |
|  | **Estimate** | **IRR/OR** | **95% CI** |
| *Count-response component* |  |  |  |
| Time | .13 | 1.14*** | (1.06 / 1.22) |
| Age | .11 | 1.11* | (1.00 / 1.23) |
| Sex: Females | -.24 | 0.78** | (0.67 / 0.92) |
| Education (in years) | .03 | 1.03 | (0.92 / 1.15) |
| Marital Status: Single/Divorced | .06 | 1.07 | (0.86 / 1.31) |
| Marital Status: Widowed | .01 | 1.01 | (0.84 / 1.22) |
| Depressive Symptoms | .06 | 1.06 | (0.98 / 1.15) |
| Medicare Health Insurance: No | -.18 | 0.84 | (0.66 / 1.05) |
| Self-rated Health: Poor | .17 | 1.19 | (0.93 / 1.52) |
| Objective Health: Poor | .54 | 1.70*** | (1.42 / 2.05) |
| Health Asymmetry | -.21 | 0.81** | (0.70 / 0.92) |
| *Zero-response component* |  |  |  |
| Time | -.02 | 0.98 | (0.70 / 1.27) |
| Age | -.82 | 0.44* | (0.22 / 0.85) |
| Sex: Females | .14 | 0.87 | (0.48 / 1.56) |
| Education (in years) | -.43 | 0.65* | (0.45 / 0.94) |
| Marital Status: Single/Divorced | .07 | 1.07 | (0.52 / 2.17) |
| Marital Status: Widowed | -.51 | 0.60 | (0.20 / 1.83) |
| Depressive Symptoms | -.33 | 0.72 | (0.51 / 1.01) |
| Medicare Health Insurance: No | .04 | 1.04 | (0.54 / 2.03) |
| Self-rated Health: Poor | -.24 | 1.27 | (0.54 / 3.02) |
| Objective Health: Poor | -.17 | 0.84 | (0.42 / 1.71) |
| Health Asymmetry | .37 | 1.44 | (0.89 / 2.33) |
| *Model fit and random components* |  |  |  |
| AIC | 17688.18 |  |  |
| BIC | 17848.18 |  |  |
| Log-likelihood | -8818.18 |  |  |
| Random Intercept Variance | .88 |  |  |
| Dispersion Parameter | 1.72 |  |  |
| Frequency (Proportion) of Zeros | 9,538 (68.6%) |  |  |
| Notes: IRR: Incidence Rate Ratio; OR: Odds Ratio; AIC: Akaike Information Criteria; BIC: Bayesian Information Criteria; Significant at ∗*P* < .05, ∗∗*P* < .01, ∗∗∗ *P* < .001; All continuous variables were Z-standardised prior to modelling. | | | |

| **Supplementary Materials 4**  Zero-inflated Negative Binomial Mixed-effects regression models for overdispersed counts of self-reported overnight hospitalisations in older adults, with **health asymmetry modelled as non-linear** (n=4,373). | | | |
| --- | --- | --- | --- |
|  | **Estimate** | **IRR/OR** | **95% CI** |
| *Count-response component* |  |  |  |
| Time | .13 | 1.13*** | (1.07 / 1.20) |
| Age | .09 | 1.09* | (1.02 / 1.18) |
| Sex: Females | -.18 | 0.84** | (0.74 / 0.94) |
| Education (in years) | .05 | 1.05 | (0.97 / 1.14) |
| Marital Status: Single/Divorced | .05 | 1.05 | (0.91 / 1.22) |
| Marital Status: Widowed | .01 | 1.01 | (0.87 / 1.17) |
| Depressive Symptoms | .09 | 1.09** | (1.03 / 1.16) |
| Medicare Health Insurance: No | -.25 | 0.78* | (0.65 / 0.93) |
| Self-rated Health: Poor | .28 | 1.33** | (1.07 / 1.66) |
| Objective Health: Poor | .64 | 1.91*** | (1.66 / 2.18) |
| Health Asymmetry | -.26 | 0.77*** | (0.69 / 0.85) |
| Health Asymmetry^2^ | -.03 | 0.97 | (0.92 / 1.02) |
| *Zero-response component* |  |  |  |
| Time | .03 | 1.30* | (1.04 / 1.65) |
| Age | -.62 | 0.54** | (0.36 / 0.80) |
| Sex: Females | -.19 | 0.82 | (0.53 / 1.27) |
| Education (in years) | -.37 | 0.69** | (0.53 / 0.90) |
| Marital Status: Single/Divorced | -.24 | 0.47 | (0.47 / 1.32) |
| Marital Status: Widowed | -.33 | 0.36 | (0.36 / 1.43) |
| Depressive Symptoms | -.23 | 0.80* | (0.63 / 0.99) |
| Medicare Health Insurance: No | .23 | 1.26 | (0.75 / 2.13) |
| Self-rated Health: Poor | .27 | 1.31 | (0.2 / 2.76) |
| Objective Health: Poor | .09 | 1.09 | (0.64 / 1.85) |
| Health Asymmetry | .21 | 1.23 | (0.79 / 1.92) |
| Health Asymmetry^2^ | -.11 | 0.89 | (0.73 / 1.08) |
| *Model fit and random components* |  |  |  |
| AIC | 25080.36 |  |  |
| BIC | 25258.92 |  |  |
| Log-likelihood | -12512.18 |  |  |
| Random Intercept Variance | .95 |  |  |
| Dispersion Parameter | 2.23 |  |  |
| Frequency (Proportion) of Zeros | 11,290 (65%) |  |  |
| Notes: IRR: Incidence Rate Ratio; OR: Odds Ratio; AIC: Akaike Information Criteria; BIC: Bayesian Information Criteria; Significant at ∗*P* < .05, ∗∗*P* < .01, ∗∗∗ *P* < .001; All continuous variables were Z-standardised prior to modelling. | | | |

| **Supplementary Materials 5**  Zero-inflated Negative Binomial Mixed-effects regression models for overdispersed counts of self-reported overnight hospitalisations in older adults, with **age and sex interactions** with health asymmetry (n=4,373). | | | |
| --- | --- | --- | --- |
|  | **Estimate** | **IRR/OR** | **95% CI** |
| *Count-response component* |  |  |  |
| Time | .14 | 1.15*** | (1.10 / 1.21) |
| Age | .10 | 1.11** | (1.03 / 1.20) |
| Sex: Females | -.16 | 0.84** | (0.75 / 0.95) |
| Education (in years) | .07 | 1.07 | (1.00 / 1.15) |
| Marital Status: Single/Divorced | .05 | 1.05 | (0.92 / 1.21) |
| Marital Status: Widowed | .01 | 1.01 | (0.88 / 1.16) |
| Depressive Symptoms | .09 | 1.10** | (1.04 / 1.16) |
| Medicare Health Insurance: No | -.25 | 0.78** | (0.65 / 0.93) |
| Self-rated Health: Poor | .21 | 1.23* | (1.01 / 1.49) |
| Objective Health: Poor | .68 | 1.97*** | (1.74 / 2.24) |
| Health Asymmetry | -.31 | 0.73*** | (0.66 / 0.82) |
| Health Asymmetry*Age | .04 | 1.04 | (0.99 / 1.10) |
| Health Asymmetry*Sex:Female | -.05 | 0.95 | (0.85 / 1.06) |
| *Zero-response component* |  |  |  |
| Time | .42 | 1.51** | (1.19 / 1.93) |
| Age | -.75 | 0.47** | (0.32 / 0.71) |
| Sex: Females | -.14 | 0.87 | (0.54 / 1.40) |
| Education (in years) | -.32 | 0.73** | (0.57 / 0.92) |
| Marital Status: Single/Divorced | -.22 | 0.81 | (0.49 / 1.31) |
| Marital Status: Widowed | -.36 | 0.70 | (0.37 / 1.31) |
| Depressive Symptoms | -.17 | 0.84 | (0.68 / 1.04) |
| Medicare Health Insurance: No | .24 | 1.27 | (0.73 / 2.21) |
| Self-rated Health: Poor | -.13 | 0.88 | (0.42 / 1.83) |
| Objective Health: Poor | .35 | 1.42 | (0.85 / 2.38) |
| Health Asymmetry | -.35 | 0.71 | (0.44 / 1.13) |
| Health Asymmetry*Age | -.43 | 0.65** | (0.48 / 0.87) |
| Health Asymmetry*Sex:Female | .04 | 1.05 | (0.67 / 1.61) |
| *Model fit and random components* |  |  |  |
| AIC | 25066.75 |  |  |
| BIC | 25258.06 |  |  |
| Log-likelihood | -12503.38 |  |  |
| Random Intercept Variance | .95 |  |  |
| Dispersion Parameter | 2.18 |  |  |
| Frequency of Zeros | 11,290 (65%) |  |  |
| Notes: IRR: Incidence Rate Ratio; OR: Odds Ratio; AIC: Akaike Information Criteria; BIC: Bayesian Information Criteria; Significant at ∗*P* < .05, ∗∗*P* < .01, ∗∗∗ *P* < .001; All continuous variables were Z-standardised prior to modelling. | | | |

**Supplementary Materials 6**

\# -------------------------

\# Objective Physical Health Factor Analysis

\# -------------------------

\# sex- and height-adjusted physical health scores

\# peak expiratory flow

pef_adj <- resid(lm(pef_mean ~ sex + height, data = df))

\# grip strength

grip_adj <- resid(lm(grip_mean ~ sex + height, data = df))

\# gait speed

walk_adj <- resid(lm(walk_mean ~ sex + height, data = df))

df$pef_adj <- pef_adj

df$grip_adj <- grip_adj

df$walk_adj <- walk_adj

\# standardise scores

df$pef_std	<- scale(df$pef_adj)

df$grip_std	<- scale(df$grip_adj)

df$walk_std	<- -scale(df$walk_adj)

df$disease_std <- -scale(df$disease)

health_variables <- df[, c("pef_std", "grip_std", "walk_std", "disease_std")]

#factor analysis

fa_result <- fa(health_variables, nfactors = 1, scores = "regression")

df$latent_health <- fa_result$scores[, 1]

\# extract eigenvalues

eigenvalues <- fa_result$e.values

print("Eigenvalues:")

print(eigenvalues)

\# extract factor loadings

loadings <- fa_result$loadings

print("Factor Loadings:")

print(loadings)

\# calculate Cronbach's alpha

alpha_result <- alpha(health_vars)

print("Cronbach's Alpha:")

print(alpha_result$total$raw_alpha)

\# variance explained by the first factor

variance_explained <- (eigenvalues[1] / sum(eigenvalues)) * 100

print(paste("Variance explained by Factor 1:", round(variance_explained, 2), "%"))

print("Factor Analysis Summary:")

print(fa_result)

\# -------------------------

\# Health Asymmetry

\# -------------------------

model = lm(srh ~ ph, data = df)

df$healthasymmetry = resid(model)

\# -------------------------

\# Zero-inflated negative binomial mixed-effects models

\# -------------------------

library(GLMMadaptive)

\# Standardise continuous variables

vars_z = c("education", "age", "cesd", "time")

df[ paste0(vars_z, "_z") ] = scale(df[vars_z])

\# -------------------------

\# Model 1: Subjective health

\# -------------------------

fm1 <- mixed_model(

fixed = hospital_count ~ time_z + age_z + sex + education_z + marriage + cesd_z + medicare + srh_group,

zi_fixed = hospital_count ~ time_z + age_z + sex + education_z + marriage + cesd_z + medicare + srh_group,

random = ~ 1 | HHIDPN,

data = df,

family = zi.negative.binomial())

summary(fm1)

exp(confint(fm1))

z1 <- confint(fm1, parm = "zero_part")

exp(z1)

\# -------------------------

\# Model 2: Physical health

\# -------------------------

fm2 <- mixed_model(

fixed = hospital_count ~ time_z + age_z + sex + education_z + marriage + cesd_z + medicare + ph_group,

zi_fixed = hospital_count ~ time_z + age_z + sex + education_z + marriage + cesd_z + medicare + ph_group,

random = ~ 1 | HHIDPN,

data = df,

family = zi.negative.binomial())

\# -------------------------

\# Model 3: SH and PH interaction

\# -------------------------

fm3 <- mixed_model(

fixed = hospital_count ~ time_z + age_z + sex + education_z + marriage + cesd_z + medicare + srh_group*ph_group,

zi_fixed = hospital_count ~ time_z + age_z + sex + education_z + marriage + cesd_z + medicare + srh_group*ph_group,

data = df,

family = zi.negative.binomial())

\# -------------------------

\# Model 4: Health asymmetry

\# -------------------------

fm4 <- mixed_model(

fixed = hospital_count ~ time_z + age_z + sex + education_z + marriage + cesd_z + medicare + healthasymmetry,

zi_fixed = hospital_count ~ time_z + age_z + sex + education_z + marriage + cesd_z + medicare + healthasymmetry,

random = ~ 1 | HHIDPN,

data = df,

family = zi.negative.binomial())

\# -------------------------

\# Model 5: Asymmetry + covariates

\# -------------------------

fm5 <- mixed_model(

fixed = hospital_count ~ time_z + age_z + sex + education_z + marriage + cesd_z + medicare + healthasymmetry + srh_group + ph_group,

zi_fixed = hospital_count ~ time_z + age_z + sex + education_z + marriage + cesd_z + medicare + healthasymmetry + srh_group + ph_group,

random = ~ 1 | HHIDPN,

data = df,

family = zi.negative.binomial())

\# -------------------------

\# Sensitivity: Nonlinear asymmetry

\# -------------------------

fm_nl <- mixed_model(

fixed = hospital_count ~ time_z + age_z + sex + education_z + marriage + cesd_z + medicare + healthasymmetry + I(healthasymmetry^2) + srh_group + ph_group,

zi_fixed = hospital_count ~ time_z + age_z + sex + education_z + marriage + cesd_z + medicare + healthasymmetry I(healthasymmetry^2) + srh_group + ph_group,

random = ~ 1 | HHIDPN,

data = df,

family = zi.negative.binomial())

\# -------------------------

\# Sensitivity: Age & sex interactions

\# -------------------------

fm_int <- mixed_model(

fixed = hospital_count ~ time_z + age_z + sex + education_z + marriage + cesd_z + medicare + healthasymmetry*age + healthasymmetry*sex + srh_group + ph_group,

zi_fixed = hospital_count ~ time_z + age_z + sex + education_z + marriage + cesd_z + medicare + healthasymmetry*age + healthasymmetry*sex + srh_group + ph_group,

random = ~ 1 | HHIDPN,

data = df,

family = zi.negative.binomial())

\# -------------------------

\# Sensitivity: Reverse Causality

\# -------------------------

fm_rev <- mixed_model(

fixed = hospital_count ~ time_z + age_z + sex + education_z + marriage + cesd_z + medicare + healthasymmetry + srh_group + ph_group,

zi_fixed = hospital_count ~ time_z + age_z + sex + education_z + marriage + cesd_z + medicare + healthasymmetry + srh_group + ph_group,

random = ~ 1 | HHIDPN,

data = df_removed,

family = zi.negative.binomial())

**Supplementary Materials 7**

Distribution of subjective health, objective health and health asymmetry scores.
